# Diaqua­[*N*-(5-nitro-2-oxidobenzyl­idene)glycinato]copper(II) dihydrate

**DOI:** 10.1107/S1600536810010652

**Published:** 2010-03-27

**Authors:** Yang Zou, Yin-Zhi Jiang, Wei-Zu Wang

**Affiliations:** a Chemistry Department, Zhejiang Sci-Tech University, Hangzhou 310018, People’s Republic of China

## Abstract

In the title complex, [Cu(C_9_H_6_N_2_O_5_)(H_2_O)_2_]·2H_2_O, the Cu^II^ atom has a square-pyramidal coordination environment with a tridentate *N*-(5-nitro-2-oxidobenzyl­idene)glycinate Schiff base ligand and a water mol­ecule in the basal plane. The apical site is occupied by an O atom from another coordinated water mol­ecule. The crystal structure is stabilized by O—H⋯O hydrogen bonds, building a two-dimensional network parallel to (100).

## Related literature

For general background to metabolic reactions requiring pyridoxal-5′-phosphate as a cofactor, see: Bkouche-Waksman *et al.* (1988 ▶); Wetmore *et al.* (2001 ▶); Zabinski & Toney (2001 ▶). For related Schiff base complexes, see: Ganguly *et al.* (2008 ▶); Jammi *et al.* (2008 ▶). For a related structure, see: Ueki *et al.* (1967 ▶).
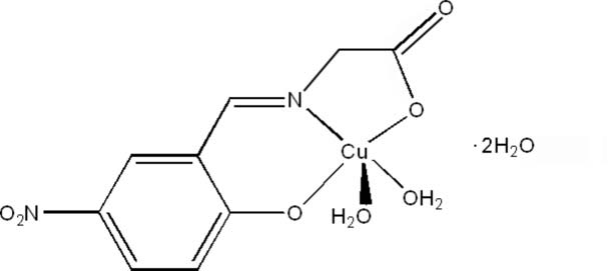


## Experimental

### 

#### Crystal data

[Cu(C_9_H_6_N_2_O_5_)(H_2_O)_2_]·2H_2_O
*M* *_r_* = 357.76
Monoclinic, 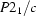

*a* = 17.306 (4) Å
*b* = 10.837 (2) Å
*c* = 7.185 (2) Å
β = 91.63 (1)°
*V* = 1347.0 (5) Å^3^
*Z* = 4
Mo *K*α radiation
μ = 1.67 mm^−1^
*T* = 293 K
0.25 × 0.20 × 0.15 mm

#### Data collection

Bruker SMART 1000 CCD diffractometer
Absorption correction: multi-scan (*SADABS*; Sheldrick, 1996 ▶) *T*_min_ = 0.68, *T*_max_ = 0.78
6554 measured reflections
2369 independent reflections
1107 reflections with *I* > 2σ(*I*)
*R*_int_ = 0.122

#### Refinement

*R*[*F* ^2^ > 2σ(*F* ^2^)] = 0.045
*wR*(*F* ^2^) = 0.074
*S* = 0.62
2369 reflections
214 parameters
8 restraints
H atoms treated by a mixture of independent and constrained refinement
Δρ_max_ = 0.53 e Å^−3^
Δρ_min_ = −0.41 e Å^−3^

### 

Data collection: *SMART* (Bruker, 2007 ▶); cell refinement: *SAINT* (Bruker, 2007 ▶); data reduction: *SAINT*; program(s) used to solve structure: *SHELXTL* (Sheldrick, 2008 ▶); program(s) used to refine structure: *SHELXTL*; molecular graphics: *SHELXTL*; software used to prepare material for publication: *SHELXTL* and *DIAMOND* (Brandenburg, 1999 ▶).

## Supplementary Material

Crystal structure: contains datablocks I, global. DOI: 10.1107/S1600536810010652/hy2290sup1.cif

Structure factors: contains datablocks I. DOI: 10.1107/S1600536810010652/hy2290Isup2.hkl

Additional supplementary materials:  crystallographic information; 3D view; checkCIF report

## Figures and Tables

**Table 1:** Hydrogen-bond geometry (Å, °)

*D*—H⋯*A*	*D*—H	H⋯*A*	*D*⋯*A*	*D*—H⋯*A*
O6—H6*A*⋯O7	0.80 (4)	1.90 (4)	2.666 (5)	162 (5)
O6—H6*B*⋯O9^i^	0.80 (3)	1.96 (3)	2.726 (5)	162 (5)
O7—H7*A*⋯O3^ii^	0.85 (3)	1.90 (3)	2.749 (4)	178 (4)
O7—H7*B*⋯O9^iii^	0.85 (3)	2.03 (4)	2.814 (5)	154 (4)
O8—H8*D*⋯O1^iv^	0.82 (3)	2.05 (3)	2.860 (4)	167 (5)
O8—H8*E*⋯O7	0.85 (4)	1.96 (4)	2.792 (5)	166 (4)
O9—H9*B*⋯O8^v^	0.84 (3)	2.04 (2)	2.827 (5)	156 (5)
O9—H9*D*⋯O2^iv^	0.78 (3)	1.99 (3)	2.764 (5)	173 (5)

Symmetry codes: (i) 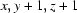; (ii) 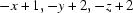; (iii) 
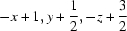
; (iv) 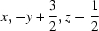; (v) 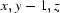.

**Fig. 1.**
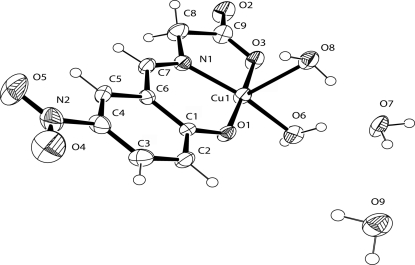
Molecular structure of the title complex. Displacement ellipsoids are drawn at the 40% probability level.

**Fig. 2.**
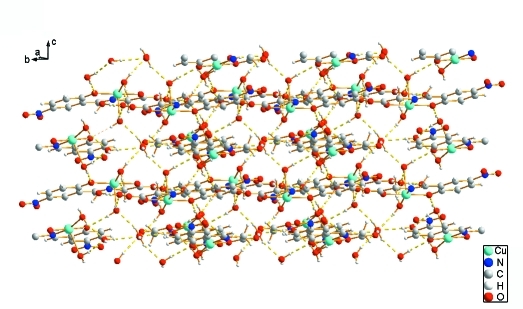
A view of the hydrogen-bonded (dashed lines) two-dimensional network.

## supplementary crystallographic information

### Comment

Metal Schiff-base complexes derived from amino acids (or peptides) play an important role as key compounds for modeling more complicated PLP-amino acid Schiff bases (PLP = pyridoxal-5'-phosphate), as these are key intermediates in a variety of metabolic reactions involving amino acids, such as decarboxylation, transamination, racemization and C—C bond cleavage, which are catalyzed by enzymes that require PLP as a cofactor (Bkouche-Waksman *et al.*, 1988; Wetmore *et al.*, 2001; Zabinski & Toney, 2001). Considerable effort has been devoted to the preparation, structural characterization, appropriate spectroscopy and magnetic studies of Schiff-base complexes derived from salicylaldehyde and amino acids and reduced salicylidene amino acid (Ganguly *et al.*, 2008), but little attention has been given to Schiff bases derived from nitro-substituted salicylaldehyde, and few structurally characterized complexes have been reported (Jammi *et al.*, 2008). Herein, we report the synthesis and structural study of a copper(II) complex with the Schiff base derived from glycine and 5-nitrosalicylaldehyde.

The title complex is characterized by a square-pyramidal Cu^II^ coordination with a deprotonated tridentate Schiff base, 5-nitrosalicylideneglycinate, and a water molecule in the basal plane (Fig. 1). The Cu—N bond distance is 1.897 (4) Å. The two Cu—O bonds are 1.916 (3) and 1.930 (3) Å. The apical Cu1—O8 bond length is 2.447 (4) Å, which is little longer than that in the parent compound of this structure type, aqua(N-salicylideneglycinato)copper(II) hemihydrate [2.334 (6) Å] (Ueki *et al.*, 1967). The phenyl ring (C1–C6) and the C1, C6, C7, N1, O1, Cu1 chelating ring are almost coplanar, with a small dihedral angle of 4.3 (4)°. Hydrogen bonds between the coordinated water molecules and the phenol O atoms of symmetry-related complex molecules lead to the formation of zigzag chains along the *c* axis (Table 1). Hydrogen bonds between the uncoordinated water molecules and carbonylate O atoms link the chains into a two-dimensional layer (Fig. 2).

### Experimental

The title compound was prepared as follows: Glycine (10 mmol), 5-nitrosalicylaldehyde (10 mmol) and LiOH (20 mmol) were dissolved and refluxed in MeOH/H_2_O (v/v 1:1). CuCl_2_.2H_2_O (10 mmol) was then added to the solution and the resulting solution was adjusted to pH = 9–11. The mixture was stirred at room temperature for 24 h. Violet-blue precipitate that formed was filtered. The filtrate was allowed to evaporate slowly at room temperature. After several days, blue crystals suitable for X-ray diffraction were obtained.

### Refinement

H atoms of water molecules were located in a difference Fourier map and refined with distance restraints of O—H = 0.85 (1) Å, and with *U*_iso_(H) = 1.2*U*_eq_(O). Other H atoms were positioned geometrically and refined using a riding model, with C—H = 0.93 (CH) and 0.97 (CH_2_) Å and with *U*_iso_(H) = 1.2*U*_eq_(C).

### Crystal data

**Table:**

[Cu(C_9_H_6_N_2_O_5_)(H_2_O)_2_]·2H_2_O	*F*(000) = 732
*M**_r_* = 357.76	*D*_x_ = 1.764 Mg m^−^^3^
Monoclinic, *P*2_1_/*c*	Mo *K*α radiation, λ = 0.71073 Å
Hall symbol: -P 2ybc	Cell parameters from 1107 reflections
*a* = 17.306 (4) Å	θ = 2.2–25.0°
*b* = 10.837 (2) Å	µ = 1.67 mm^−^^1^
*c* = 7.185 (2) Å	*T* = 293 K
β = 91.63 (1)°	Block, blue
*V* = 1347.0 (5) Å^3^	0.25 × 0.2 × 0.15 mm
*Z* = 4	

### Data collection

**Table:**

Bruker SMART 1000 CCD diffractometer	2369 independent reflections
Radiation source: fine-focus sealed tube	1107 reflections with *I* > 2σ(*I*)
graphite	*R*_int_ = 0.122
φ and ω scans	θ_max_ = 25.0°, θ_min_ = 2.2°
Absorption correction: multi-scan (*SADABS*; Sheldrick, 1996)	*h* = −20→20
*T*_min_ = 0.68, *T*_max_ = 0.78	*k* = −8→12
6554 measured reflections	*l* = −8→8

### Refinement

**Table:**

Refinement on *F*^2^	Primary atom site location: structure-invariant direct methods
Least-squares matrix: full	Secondary atom site location: difference Fourier map
*R*[*F*^2^ > 2σ(*F*^2^)] = 0.045	Hydrogen site location: inferred from neighbouring sites
*wR*(*F*^2^) = 0.074	H atoms treated by a mixture of independent and constrained refinement
*S* = 0.62	*w* = 1/[σ^2^(*F*_o_^2^)] where *P* = (*F*_o_^2^ + 2*F*_c_^2^)/3
2369 reflections	(Δ/σ)_max_ < 0.001
214 parameters	Δρ_max_ = 0.53 e Å^−^^3^
8 restraints	Δρ_min_ = −0.41 e Å^−^^3^

### Fractional atomic coordinates and isotropic or equivalent isotropic displacement parameters (Å^2^)

**Table:**

	*x*	*y*	*z*	*U*_iso_*/*U*_eq_	
Cu1	0.68418 (3)	0.93382 (5)	1.05603 (9)	0.0332 (2)	
N1	0.7825 (2)	0.9810 (4)	0.9699 (6)	0.0313 (11)	
N2	0.9642 (3)	0.5241 (5)	0.7898 (7)	0.0533 (15)	
C1	0.7707 (3)	0.7116 (5)	1.0012 (7)	0.0262 (13)	
C2	0.7740 (3)	0.5812 (4)	0.9948 (7)	0.0342 (14)	
H2	0.7324	0.5352	1.0357	0.041*	
C3	0.8371 (3)	0.5222 (5)	0.9296 (7)	0.0384 (15)	
H3	0.8381	0.4364	0.9284	0.046*	
C4	0.8991 (3)	0.5861 (5)	0.8659 (7)	0.0362 (14)	
C5	0.8984 (3)	0.7134 (5)	0.8696 (7)	0.0326 (14)	
H5	0.9409	0.7572	0.8285	0.039*	
C6	0.8343 (2)	0.7767 (4)	0.9347 (7)	0.0241 (12)	
C7	0.8375 (2)	0.9109 (4)	0.9286 (6)	0.0319 (14)	
H7	0.8832	0.9473	0.8917	0.038*	
C8	0.7918 (3)	1.1165 (4)	0.9543 (8)	0.0452 (16)	
H8A	0.8051	1.1380	0.8281	0.054*	
H8B	0.8334	1.1440	1.0377	0.054*	
C9	0.7174 (3)	1.1797 (5)	1.0040 (8)	0.0404 (15)	
O1	0.70921 (17)	0.7616 (3)	1.0634 (5)	0.0314 (9)	
O2	0.71569 (18)	1.2917 (3)	0.9976 (5)	0.0533 (12)	
O3	0.66318 (17)	1.1087 (3)	1.0507 (5)	0.0405 (10)	
O4	0.9629 (2)	0.4122 (3)	0.7780 (6)	0.0807 (16)	
O5	1.0193 (2)	0.5843 (4)	0.7388 (6)	0.0828 (16)	
O6	0.59324 (19)	0.9035 (4)	1.2066 (5)	0.0389 (10)	
H6A	0.559 (3)	0.890 (5)	1.134 (5)	0.053*	
H6B	0.583 (3)	0.942 (4)	1.297 (5)	0.047*	
O7	0.48642 (17)	0.8064 (3)	0.9756 (6)	0.0466 (11)	
H7A	0.4407 (11)	0.834 (3)	0.966 (7)	0.053*	
H7B	0.481 (2)	0.729 (3)	0.982 (7)	0.053*	
O8	0.6090 (2)	0.8942 (3)	0.7687 (6)	0.0403 (10)	
H8E	0.5724 (19)	0.857 (3)	0.821 (6)	0.053*	
H8D	0.631 (2)	0.844 (4)	0.703 (6)	0.048*	
O9	0.58080 (19)	0.0716 (3)	0.4864 (5)	0.0438 (10)	
H9D	0.621 (2)	0.105 (4)	0.486 (7)	0.053*	
H9B	0.576 (2)	0.026 (4)	0.580 (4)	0.053*	

### Atomic displacement parameters (Å^2^)

**Table:**

	*U*^11^	*U*^22^	*U*^33^	*U*^12^	*U*^13^	*U*^23^
Cu1	0.0270 (3)	0.0226 (3)	0.0505 (4)	0.0012 (3)	0.0084 (3)	0.0002 (4)
N1	0.028 (2)	0.023 (3)	0.044 (3)	0.006 (2)	0.005 (2)	0.004 (2)
N2	0.039 (3)	0.058 (4)	0.064 (4)	0.024 (3)	0.009 (3)	0.000 (3)
C1	0.022 (3)	0.029 (3)	0.028 (3)	−0.001 (3)	0.003 (3)	0.006 (3)
C2	0.027 (3)	0.027 (3)	0.049 (4)	−0.001 (3)	0.011 (3)	0.007 (3)
C3	0.045 (4)	0.035 (3)	0.036 (4)	0.004 (3)	0.008 (3)	0.003 (3)
C4	0.038 (3)	0.033 (4)	0.038 (4)	0.015 (3)	0.005 (3)	0.000 (3)
C5	0.021 (3)	0.038 (4)	0.039 (4)	−0.004 (3)	0.004 (3)	0.001 (3)
C6	0.023 (3)	0.022 (3)	0.027 (3)	0.001 (2)	0.000 (3)	−0.002 (3)
C7	0.025 (3)	0.029 (3)	0.041 (4)	−0.005 (3)	0.005 (3)	0.001 (3)
C8	0.043 (4)	0.026 (3)	0.067 (5)	−0.003 (3)	0.011 (3)	0.005 (3)
C9	0.038 (4)	0.033 (4)	0.051 (4)	0.000 (3)	0.005 (3)	−0.010 (3)
O1	0.026 (2)	0.020 (2)	0.048 (3)	−0.0014 (15)	0.0092 (19)	0.0026 (17)
O2	0.049 (3)	0.016 (2)	0.096 (3)	−0.0022 (19)	0.011 (2)	−0.005 (2)
O3	0.031 (2)	0.018 (2)	0.073 (3)	−0.0009 (16)	0.010 (2)	−0.0023 (19)
O4	0.077 (3)	0.037 (3)	0.130 (4)	0.025 (2)	0.026 (3)	−0.013 (3)
O5	0.048 (3)	0.068 (3)	0.135 (4)	0.022 (3)	0.047 (3)	−0.003 (3)
O6	0.037 (2)	0.036 (3)	0.044 (3)	0.0001 (19)	0.007 (2)	−0.008 (2)
O7	0.027 (2)	0.035 (2)	0.079 (3)	−0.0045 (19)	0.008 (2)	−0.001 (2)
O8	0.039 (2)	0.029 (2)	0.053 (3)	0.0002 (17)	0.008 (2)	0.000 (2)
O9	0.045 (2)	0.032 (2)	0.056 (3)	−0.004 (2)	0.010 (2)	0.001 (2)

### Geometric parameters (Å, °)

**Table:**

Cu1—N1	1.897 (4)	C5—C6	1.396 (6)
Cu1—O1	1.916 (3)	C5—H5	0.9300
Cu1—O3	1.930 (3)	C6—C7	1.456 (6)
Cu1—O6	1.963 (4)	C7—H7	0.9300
Cu1—O8	2.447 (4)	C8—C9	1.511 (6)
N1—C7	1.260 (5)	C8—H8A	0.9700
N1—C8	1.481 (5)	C8—H8B	0.9700
N2—O4	1.215 (5)	C9—O2	1.215 (5)
N2—O5	1.221 (5)	C9—O3	1.266 (5)
N2—C4	1.433 (6)	O6—H6B	0.80 (3)
C1—O1	1.286 (5)	O6—H6A	0.80 (4)
C1—C6	1.404 (6)	O7—H7A	0.85 (3)
C1—C2	1.415 (6)	O7—H7B	0.85 (3)
C2—C3	1.360 (6)	O8—H8D	0.82 (3)
C2—H2	0.9300	O8—H8E	0.85 (4)
C3—C4	1.367 (6)	O9—H9D	0.78 (3)
C3—H3	0.9300	O9—H9B	0.84 (3)
C4—C5	1.380 (6)		
			
N1—Cu1—O1	93.87 (15)	C5—C4—N2	118.9 (5)
N1—Cu1—O3	84.20 (15)	C4—C5—C6	120.3 (5)
O1—Cu1—O3	177.76 (14)	C4—C5—H5	119.8
N1—Cu1—O6	164.91 (16)	C6—C5—H5	119.8
O1—Cu1—O6	90.32 (15)	C5—C6—C1	120.4 (5)
O3—Cu1—O6	91.26 (15)	C5—C6—C7	116.8 (4)
N1—Cu1—O8	103.44 (14)	C1—C6—C7	122.9 (4)
O1—Cu1—O8	88.09 (13)	N1—C7—C6	124.5 (4)
O3—Cu1—O8	93.45 (13)	N1—C7—H7	117.7
O6—Cu1—O8	91.16 (14)	C6—C7—H7	117.7
C7—N1—C8	119.7 (4)	N1—C8—C9	109.6 (4)
C7—N1—Cu1	127.2 (3)	N1—C8—H8A	109.7
C8—N1—Cu1	113.1 (3)	C9—C8—H8A	109.7
O4—N2—O5	121.6 (5)	N1—C8—H8B	109.7
O4—N2—C4	118.8 (5)	C9—C8—H8B	109.7
O5—N2—C4	119.5 (5)	H8A—C8—H8B	108.2
O1—C1—C6	124.8 (5)	O2—C9—O3	126.9 (5)
O1—C1—C2	117.9 (4)	O2—C9—C8	117.6 (5)
C6—C1—C2	117.3 (5)	O3—C9—C8	115.5 (5)
C3—C2—C1	120.9 (5)	C1—O1—Cu1	126.1 (3)
C3—C2—H2	119.5	C9—O3—Cu1	117.5 (3)
C1—C2—H2	119.5	Cu1—O6—H6B	125 (4)
C2—C3—C4	121.5 (5)	Cu1—O6—H6A	106 (3)
C2—C3—H3	119.2	H6B—O6—H6A	116 (4)
C4—C3—H3	119.2	H7A—O7—H7B	105 (3)
C3—C4—C5	119.5 (5)	H8D—O8—H8E	108 (3)
C3—C4—N2	121.5 (5)	H9D—O9—H9B	112 (4)

### Hydrogen-bond geometry (Å, °)

**Table:**

*D*—H···*A*	*D*—H	H···*A*	*D*···*A*	*D*—H···*A*
O6—H6A···O7	0.80 (4)	1.90 (4)	2.666 (5)	162 (5)
O6—H6B···O9^i^	0.80 (3)	1.96 (3)	2.726 (5)	162 (5)
O7—H7A···O3^ii^	0.85 (3)	1.90 (3)	2.749 (4)	178 (4)
O7—H7B···O9^iii^	0.85 (3)	2.03 (4)	2.814 (5)	154 (4)
O8—H8D···O1^iv^	0.82 (3)	2.05 (3)	2.860 (4)	167 (5)
O8—H8E···O7	0.85 (4)	1.96 (4)	2.792 (5)	166 (4)
O9—H9B···O8^v^	0.84 (3)	2.04 (2)	2.827 (5)	156 (5)
O9—H9D···O2^iv^	0.78 (3)	1.99 (3)	2.764 (5)	173 (5)

Symmetry codes: (i) *x*, *y*+1, *z*+1; (ii) −*x*+1, −*y*+2, −*z*+2; (iii) −*x*+1, *y*+1/2, −*z*+3/2; (iv) *x*, −*y*+3/2, *z*−1/2; (v) *x*, *y*−1, *z*.
